# Chronic Immune Thrombocytopenia and Hashimoto's Hypothyroidism in an Adolescent: Presentation and Implications

**DOI:** 10.1155/2021/6649155

**Published:** 2021-02-01

**Authors:** Judy Ibrahim, Mohammad Alashqar, Shamma Al Zaabi, Omar Trad, Amar Al Shibli

**Affiliations:** ^1^Academic Affairs Department, Tawam Hospital, Al Ain, UAE; ^2^Hematology-Oncology Department, Tawam Hospital, Al Ain, UAE; ^3^General Pediatrics Department, Tawam Hospital, Al Ain, UAE

## Abstract

Immune thrombocytopenia (ITP) is a disorder characterized by immune-mediated destruction of thrombocytes leading to peripheral blood platelet count of <100 × 10^9/L. Primary ITP is a terminology used in the absence of other causes or disorders that may be associated with thrombocytopenia, i.e., isolated thrombocytopenia. The term secondary ITP is used if such diseases coexist. We present here a case of a 14-year-old female diagnosed with immune thrombocytopenia. When her evaluation was not strongly supportive of primary ITP, she was screened and proved to have a concomitant Hashimoto thyroiditis. Contrary to the popular belief about secondary ITP in adult population, treatment of our patient's hypothyroidism did not improve her platelet's count, and the patient needed multiple immunosuppressive medications to improve her condition.

## 1. Introduction

Immune thrombocytopenia (ITP) is a pathology mediated by autoantibodies targeting platelet membrane glycoproteins. Antigens triggering autoantibodies are frequently present on GPIIb–IIIa or GPIb–IX complexes [1]. It could be acute or chronic if persisted for more than 12 months. Immune thrombocytopenia is said to be related to a complex interaction between environmental and genetic factors. Recent studies identified the regulatory B cells (Breg), as an immature subset of B cells that inhibit T cell activation through secretion of the anti-inflammatory interleukin-10 (IL-10). Hence, it regulates the autoimmune responses and promotes tolerance. It was observed that B cells of ITP patients have impaired IL-10 response after stimulation [2]. Hashimoto's thyroiditis (HT) is majorly attributed to cellular autoimmunity, but there is also evidence that extrathyroidal lymphoid tissues, including Breg cells, may contribute to the antibody production [3, 4].

## 2. Case Presentation

A 14-year-old Emirati female presented to the hospital with complaints of bruises for one week. She had recurrent little gingival bleeding after teeth brushing which stopped with moderate compression. No bleeding was reported otherwise. She never had such symptoms before, and there was no previous episode of prolonged bleeding after any previous trauma or medical procedure (e.g., venipuncture). There was no history of recent viral or bacterial illnesses. The patient denied headaches, skin rashes, oral and nasal ulcers, joint pain nor swelling, photosensitivity, and alopecia. Review of systems did not reveal other complaints apart from putting on weight more easily than before.

Her medical history was remarkable for the diagnosis of epilepsy 3 years ago. However, she was already on a tapering dose of levetiracetam which was stopped after a week from her current presentation. Family history was significant for a 9-year-old cousin with type 1 diabetes mellitus and multiple cousins who had hypothyroidism and were on treatment with levothyroxine. She had no allergies and her vaccines were up-to-date.

On examination, she was overweight with body mass index at 90^th^ centile for age and gender (*z*-score/SDS of 1.23 according to WHO charts). She had multiple scattered bruises of different ages over her 4 limbs, and the largest was about 5 cm over her left arm (the site of a recent venipuncture). There was no enanthems nor mucosal bleeding, no lymph nodes enlargement, and no abdominal organomegaly. There was a large homogeneous smooth goiter, with no nodularity noted. The rest of her systemic examination was unremarkable.

Table 1 presents her laboratory investigations. It was noted that anti-SS A antibodies were positive; however, upon questioning, she denied the history of dry eyes and difficulties in chewing nor in swallowing.

Liver function tests, hepatitis screen, and complement 3 and 4 levels were normal. Coombs test, anti-DsDNA, anticardiolipin, antimitochondrial antibodies (AMA), anti-Scl-70, anti-JO-1, and anti-Sm/RNP were all negative. Antiinsulin and antiglutamic acid decarboxylase (GAD) antibodies were not measured.

**Figure 1:**
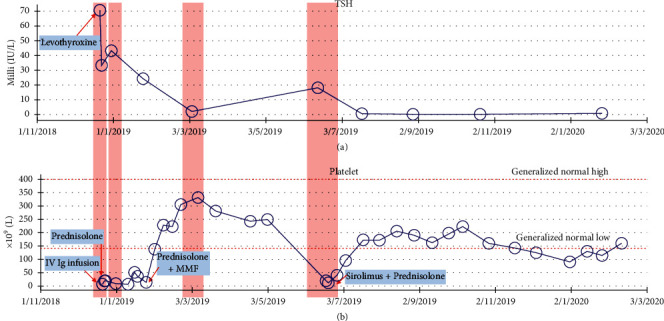
(a). Trend of TSH measurements, the level peaked twice, correlating with the times of relapses of ITP. (b). Platelets counts, note the persistent nature at the beginning of diagnosis and the frequent relapses. The timing and the type of medications used are indicated in the boxes.

She was diagnosed to have Hashimoto's thyroiditis with secondary ITP, based on clinical and laboratory parameters. The child was started on levothyroxine (50 mcg orally once a day) at presentation and was given 2 g/kg of 10% intravenous immunoglobulins (IV Ig) as an infusion according to a standardized hospital protocol. Her platelets count picked up to 19 × 10^9/L next day. After one week, the platelets count decreased to 8 × 10^9/L. At that point, 7 days course of prednisolone (1 mg/kg) was given which was effective in bringing her platelets count up to 52 × 10^9/L. 10 days later, platelets count decreased to 12 × 10^9/L. Another steroid course (1 mg/kg) of oral prednisolone was started along with mycophenolate (MMF), 1 gm orally twice daily. After a week, the platelets count increased to 137 × 10^9/L, then to 226 × 10^9/L in a week time, and steroids were started to be tapered at this level (Figure 1), with platelets checked every other week. Six weeks later, platelets decreased again. Sirolimus was started with a repeated course of steroids (similar previous dosing). The counts recovered, so the dose of sirolimus was gradually increased and steroids were gradually tapered. The child continued to require immune suppressive therapy for a year after her initial diagnosis and is still currently on sirolimus.

**Figure 2:**
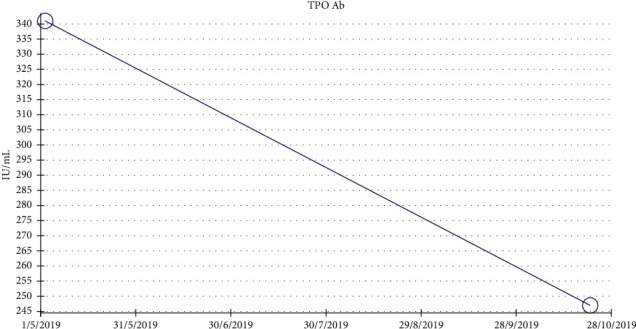
Trend of anti-TPo titers, measured every 6 months. The level was above 600 (not chartable) at first presentation. Six months later, it dropped to 340 IU/mL.

Thyroid stimulating hormone (TSH) levels decreased significantly when levothyroxine started, but it rebounded back to a slightly higher level in the second week of treatment. The dose of levothyroxine was gradually increased, and follow-up of the TSH level showed significant improvement (Figure 1). However, the significant improvement came also after starting immune suppression with steroids and MMF. Antithyroid peroxidase (anti-TPo) titer went down from being >600 (unchartable) to 247 IU/mL (Figure 2).

## 3. Discussion

Our case report highlights poly-autoimmunity and the implications of presence of thyroid autoimmune disease in the development of chronic ITP. The prevalence of antithyroid peroxidase antibodies (anti-TPo) in healthy pediatric population is estimated to be 3.4% [5], while the incident of ITP is around 1.9–6.4 cases per 100,000 per year [6]. There is probably more than a casual relationship between immune thrombocytopenia and autoimmune thyroid diseases. Although well documented in adult population, this association is not clear in pediatrics, its strength, whether both exist in a causative relationship or both exist in a background of common autoimmune disease. It is also not certain whether ITP should trigger screening for autoimmune thyroid diseases and whether the presence of ITP and antithyroid autoantibodies (ATA) together defines a certain outcome for both illnesses. In 2005, Pratt et al. published a small cohort on prevalence of ATA and antinuclear antibodies (ANA) in children coming with ITP, whether acute or chronic. Thirty-one patients were cohorted, five (16%) of the patients tested positive for ATA: 2 children with acute ITP and 3 with chronic ITP. Another five in the study population tested positive for ANA: 4 had chronic ITP. This observation indicates that children with these autoantibodies may be more likely to develop chronic ITP. The study also noted that chronic ITP was more prevalent in children of older ages (above 12 years of age). However, the sample size in this study was clearly small, which limited the ability to set a predictive value for antibody positivity for chronic ITP [7].

In 2013, Bay et al. estimated the prevalence and clinical significance of ATA in children with ITP. Antithyroid peroxidase (anti-TPo) and/or antithyroglobulin (anti-Tg) was found positive in 36.8% of patients enrolled in the study [8]. There was no significant difference of ratios of autoantibodies between acute and chronic ITP patients. In anti-TPo-positive patients, the initial mean platelet count was significantly less when compared to that of the anti-TPo-negative patients. The numbers were still significantly less after intravenous immunoglobulin treatment when compared to the autoantibodies-negative group. The study recommended screening ITP patients for such antibodies. In a similar but more recent report (2017) from Egypt, Mousa et al. studied sixty-one children with ITP, compared to seventy-five apparently healthy children (control group). Antithyroid antibodies (ATA) were significantly higher and more frequently positive in children with ITP than the control group. Additionally, ATA-positive children with ITP had significantly lower platelets count at diagnosis, after treatment with IV Ig and steroids, and after 1 month of follow up. They also had more frequent relapses [9].

Adult patients with secondary ITP and Hashimoto's thyroiditis are reported to show improvement in the platelets count after initiation of treatment with levothyroxine [10–12]. However, in our case, the child did not show improvement despite initiation of treatment with levothyroxine immediately, and despite improvement in thyroid function tests, an observation that might indicate a more complex defect in the immune system. To our knowledge, only 2 case reports are present in the pediatrics age group with similar findings to our case. Both suggested that the presence of ATA complicates the treatment of ITP [13, 14].

Giordano et al. published a recent retrospective study (2018) which also supported the observation of higher presence of ATA in patients with chronic ITP than in pediatrics population but stated that their presence does not seem to play a role as a prognostic factor for the chronicity of ITP. However, there were several weaknesses in the study: small population size, retrospectivity, and heterogeneity of the study sample [15].

Other autoimmune disorders which might cause thrombocytopenia include but not limited to rheumatoid arthritis (RA), systemic lupus erythematosus (SLE), and Sjogren syndrome.  Table 2 demonstrates criteria for diagnosis of SLE and Sjogren syndrome. Despite having some, the child did not fulfill all criteria needed to diagnose either of the disorders.

## 4. Conclusion

Most of ITP cases in the pediatric age group are isolated and acute. Pediatricians should be aware about the possibility of ITP coexisting with clinical and subclinical autoimmune thyroid diseases. The presence of the latter might complicate the condition and make it more difficult to treat. Furthermore, large studies are needed to make a definite conclusion as to whether thyroid hormones should be investigated in cases of chronic ITP.

## Figures and Tables

**Table 1:** Laboratory tests performed upon first presentation of the patient.

Test	Results	Normal range
Platelets	6 × 10^9/L	150–350
MPV	12.7 fL	6.5–10
RBCs	5.23 × 10^12/L	4.0–4.9
Hb	11.5 g/dL	12–16
Hct	0.36	0.37–0.45
MCV	69.2 fL	78–92
MCH	22 pg	33.9–35.4
WBC	6.8 × 10^9/L	4.5–13.5 × 10^9
Neutrophil count	3.6 × 10^9/L (52.9%)	1.5–6.5 × 10^9 (40–70)
Lymphocytes count	2.66 × 10^9/L (39.1%)	1.0–3.2 × 10^9 (28–48)
INR	1.06	0.97–1.30
aPTT	27	33.9–46.1
TSH	70.6 milli IU/L	0.52–5.05
Free T4	0.78 ng/dL	1.03–1.77
Anti-TPO Ab	>600 IU/mL	
ANA	Positive	
Anti-SS A Ab	Positive	

**Table 2:** Diagnostic criteria for systemic lupus erythematosus and Sjogren syndrome.

Systemic lupus erythematosus	Sjogren syndrome
*Clinical criteria*	1. Ocular symptoms: a positive response to at least one of the following questions:
(i) Acute cutaneous lupus	(i) Have you had daily, persistent, troublesome dry eyes for more than 3 months?
(ii) Chronic cutaneous lupus	(ii) Do you have a recurrent sensation of sand or gravel in the eyes?
(iii) Oral or nasal ulcers in the absence of other causes	(iii) Do you use tear substitutes more than 3 times a day?
(iv) Nonscarring alopecia	2. Oral symptoms: a positive response to at least one of the following questions:
(v) Synovitis involving 2 or more joints	(i) Have you had a daily feeling of dry mouth for more than 3 months?
(vi) Serositis in the absence of other causes	(ii) Have you had recurrently or persistently swollen salivary glands as an adult?
(vii) Renal disease	(iii) Do you frequently drink liquids to aid in swallowing dry food?
(viii) Neurologic disease	3. Ocular signs: a positive result for at least one of the following two tests:
(ix) Hemolytic anemia	Schirmer's *i* test or Rose Bengal score or another ocular dye score
(x) Neutropenia or lymphopenia	4. Histopathology showing focal lymphocytic sialadenitis
(xi) Thrombocytopenia	5. Salivary gland involvement evident by US or scintigraphy or the flow test.
*Immunologic criteria*	6. Autoantibodies (anti-Ro (SSA) or anti-La (SSB) or both)
(i) High ANA level	
(ii) High anti-dsDNA	
(iii) Positive anti-Sm	
(iv) Positive antiphospholipid antibodies	
(v) Low complement levels	
(vi) Positive direct Coombs test	
To diagnose SLE, the child must meet 4 criteria at least one clinical and one immunologic or biopsy-proven nephritis with positive ANA and anti-dsDNA.	The presence of any four of the above six criteria is sufficient to diagnose Sjogren syndrome
Petri et al. (derivation and validation of systemic lupus international collaborating clinics (SLICC) classification criteria for systemic lupus erythematosus. Arthritis Rheum 2012; 64:2677-2686.)	Vitali et al. (classification criteria for Sjögren's syndrome: a revised version of the European criteria proposed by the American-European Consensus Group. Ann Rheum Dis 2002; 61:554-558.)

## Data Availability

The reference data used to support the findings of this study are included within the article.
