# Corrigendum to “Chronic Immune Thrombocytopenia and Hashimoto's Hypothyroidism in an Adolescent: Presentation and Implications”

**DOI:** 10.1155/2021/9804326

**Published:** 2021-04-20

**Authors:** Judy Ibrahim, Mohammad Alashqar, Shamma Al Zaabi, Omar Trad, Eman Taryam Al Shamsi, Amar Al Shibli

**Affiliations:** ^1^Academic Affairs Department, Tawam Hospital, Al Ain, UAE; ^2^Hematology-Oncology Department, Tawam Hospital, Al Ain, UAE; ^3^General Pediatrics Department, Tawam Hospital, Al Ain, UAE

In the article titled “Chronic Immune Thrombocytopenia and Hashimoto's Hypothyroidism in an Adolescent: Presentation and Implications” [1], Dr. Eman Taryam Al Shamsi has been added to the author list, who contributed to the management of the patient. With the agreement of all authors, the corrected author list is shown above.

## References

[1] Ibrahim J., Alashqar M., Zaabi S. Al, Omar T., Al Shibli A. (2021). Chronic Immune Thrombocytopenia and Hashimoto’s Hypothyroidism in an Adolescent: Presentation and Implications. *Case Reports in Pediatrics*.

